# Transcriptome Profile of Rat Adrenal Evoked by Gonadectomy and Testosterone or Estradiol Replacement

**DOI:** 10.3389/fendo.2017.00026

**Published:** 2017-02-15

**Authors:** Karol Jopek, Piotr Celichowski, Marta Szyszka, Marianna Tyczewska, Paulina Milecka, Ludwik K. Malendowicz, Marcin Rucinski

**Affiliations:** ^1^Department of Histology and Embryology, Poznan University of Medical Science, Poznan, Poland

**Keywords:** rat, adrenal gland, transcriptome, global gene profiling, functional annotation clustering, gonadectomy, testosterone, estradiol

## Abstract

Sex differences in adrenal cortex structure and function are well known in different species. In the rat, they are manifested as larger adrenal cortex and higher corticosterone secretion by females compared with males. These sex differences depend, among others, on functioning of the hypothalamic-pituitary-adrenal axis (HPA). In this aspect, it is widely accepted that testosterone exerts an inhibitory and estradiol stimulatory effect on the said axis. The molecular bases of these sex-related differences are poorly understood. Therefore, we performed studies aimed to demonstrate the effect of testosterone and estradiol on the expression of differentially regulated genes in rat adrenal gland. The classical method applied in the study—gonadectomy and gonadal hormone replacement—allows obtaining results suggesting a physiological role of the tested hormone (testosterone or estradiol) in the regulation of the specific genes. Adult male and female rats were either gonadectomized or sham operated. Half of orchiectomized rats were replaced with testosterone while ovariectomized ones with estradiol. Transcriptome was identified by means of Affymetrix^®^ Rat Gene 2.1 ST Array. Differentially expressed genes were analyzed by means of DAVID web-based bioinformatic tools and confirmed by means of Gene Set Enrichment Analysis. For selected genes, validation of the results was performed using QPCR. Performed experiments have provided unexpected results. Contrary to expectations, in orchiectomized rats, testosterone replacement stimulates expression of numerous genes, mainly those associated with lipids and cholesterol metabolism. However, in ovariectomized animals, estradiol replacement inhibits the expression of genes, mainly those involved in intracellular signaling pathways. The physiological relevance of these findings awaits further research.

## Introduction

Sex-related differences in the structure and function of the adrenal cortex of mature rats are well recognized. These differences are the result of the action of sex hormones on the various components of the hypothalamic-pituitary-adrenal axis (HPA). The results of studies on the effects of gonadectomy and sex hormone replacement on the rat adrenal cortex suggest that estrogens stimulate and androgens inhibit the HPA axis function. The results of these studies are presented in many comprehensive monographs and textbooks (1–7). It is important to note that in gonadectomized animals sex hormones exert generalized response, not limited to HPA axis only. For example, they regulate the levels of corticosteroid-binding globulins (CBG) (8–11) as well as corticosterone biological half-time (12–14). CBG is involved in the mechanism of steroid hormone action by keeping steroids in circulation, and therefore controlling the availability of unbound hormones for their targets. In the rat, CBG levels are notably higher in females. Orchiectomy (ORX) resulted in 60–70% increase in CBG levels, while ovariectomy (OVX) had no effect (8, 15). Of interest, in the rat, estradiol effect on CBG is mediated via thyroid gland. Furthermore, biological half-life of corticosterone in adult male rats is ca. two times longer than in females (12, 16). Also, the metabolic clearance rate of corticosterone is markedly higher in female than male rats (17, 18).

The above-described sex differences in the structure and functions of the rat adrenal cortex probably depend on differences in the gene expression of this gland. However, to our knowledge, no studies are available on the effects of estradiol or testosterone on transcriptome profile of the rat adrenal gland. Regarding this, recently we have shown that in adult rat adrenal cortex, the differentially expressed genes that are significantly enriched included genes involved in steroid hormone metabolism, and their expression levels in females were significantly higher compared with those in the male rat (19). Generally, if compared with males, in the female rats higher expression levels of genes involved directly in steroid hormone synthesis were accompanied by lower expression levels of genes regulating basal cell functions. To our knowledge, only one publication on global gene profiling refers to the effect of testosterone on the expression of genes in the adrenal gland and it relates to the mouse (20). In this research, however, authors administered testosterone into intact female mice. Comparison between females and females treated with testosterone revealed 94 differentially regulated genes, with 23 being upregulated and 71 downregulated. The observed changes in gene expression authors combine with the disappearance of the X zone of the mouse adrenal cortex. It should be emphasize that this experimental model is not a physiological one.

In the present study, to demonstrate the effect of estradiol and testosterone on the expression of differentially regulated genes in rat adrenal gland, we applied experimental model considered in endocrinology as the “gold standard.” Dual control, it means removal of the gonads and replacement with the appropriate hormone allows obtaining results suggesting a physiological role of the tested hormone (estradiol or testosterone) in the regulation of the specific genes. Performed experiments have provided unexpected results. Contrary to expectations, in orchiectomized rats, testosterone replacement stimulates expression of numerous genes, mainly associated with lipids and lipid metabolism. However, in ovariectomized animals, estradiol replacement inhibits the expression of genes, mainly those involved in intracellular signaling pathways.

In the present study, to demonstrate the effect of estradiol and testosterone on the expression of differentially regulated genes in rat adrenal gland, we applied the classical method used to study endocrine glands, it means “remove and replace” (gonadectomy and gonadal hormone replacement). Performed experiments have provided unexpected results. Contrary to expectations, in orchiectomized rats, testosterone replacement stimulates expression of numerous genes, mainly associated with lipids and lipid metabolism. However, in ovariectomized animals, estradiol replacement inhibits the expression of genes, mainly those involved in intracellular signaling pathways.

## Materials and Methods

### Animals and Experiments

Adult female and male Wistar rats (12 weeks old; body weight: 120–150 g) were obtained from the Laboratory Animals Breeding Center, Department of Toxicology, Poznan University of Medical Sciences, Poznan, Poland. Animals were kept under standardized conditions of light (14:10 h light–dark cycle, illumination onset 06.00) at constant temperature (23°C) with free access to standard food pellets and water. All experiments were carried out between 10 and 11 am. All procedures described herein were approved by the Local Ethics Committee for Animal Research (Poznan, Poland), permission number: LKE—11/2015. All possible efforts have been done to minimize the number of animals and their suffering.

Rats were gonadectomized or sham operated under ketamine (100 mg/kg, i.p.) and xylazine (10 mg/kg, i.p.) anesthesia. ORX was performed via scrotal access while OVX by two dorsolateral incisions. Fourteen days after surgery, half of ORX rats was replaced with testosterone (s.c. injection of Testoviron-Depot, Schering AG, Berlin, 5 mg/100 g body weight), while half of OVX animals with estradiol (s.c. injection of Estradiol-Depot, Jenapharm, 0.5 mg/100 g body weight). Doses of administered depo hormones were based on previous reports (21–24). It is believed that from both compounds either testosterone or estradiol are liberated slowly, providing a physiological hormone levels in gonadectomized rats. As emphasized by Schulte-Beerbühl and Nieschlag (24), increasing the dose of injected testosterone esters appears not to influence the maximal concentrations of testosterone in the blood but rather the duration of the effect. Moreover, administration of depo compounds allows to avoid the stress evoked by daily administration of the tested substances. After 2 weeks (4 weeks post surgery), rats were decapitated. Adrenal glands were collected to RNAlader and stored in −70°C for further analyses. Seminal vesicles and uteri were also collected and weighed.

### Corticosterone, Cholesterol, and Lipoproteins Detection

Serum corticosterone levels were determined by means of ELISA kit (ELISA Demeditec kit). Serum total cholesterol, lipoproteins, and triglycerides concentrations were evaluated by means of Roche Cobas Integra 400+ system.

### RNA Extraction

Total RNA was extracted from samples of entire adrenals using TRI Reagent (Sigma, St. Louis, MO, USA) and RNeasy MinElute cleanup Kit (Qiagen, Hilden, Germany). The amount of total mRNA was determined from the optical density at 260 nm, and the RNA purity was estimated using the 260/280 nm absorption ratio (higher than 1.8) (NanoDrop spectrophotometer, Thermo Scientific, ALAB, Poland). The RNA integrity and quality were checked in a Bioanalyzer 2100 (Agilent Technologies, Inc., Santa Clara, CA, USA). The resulting RNA integrity numbers were between 8.5 and 10 with an average of 9.2 (Agilent Technologies, Inc., Santa Clara, CA, USA). Each sample was diluted to the RNA concentration of 100 ng/µl, at the OD260/OD280 ratio of 1.8/2.0. From each RNA sample, 100 ng of RNA was taken for microarray experiments. The remaining amount of isolated RNA was used for RT-qPCR study.

### Microarray Expression Analysis and Statistics

The Affimetrix procedure and methods of analyzes were described previously (19, 25–27). Total RNA (100 ng) from each sample was subjected to two rounds of sense cDNA amplification (Ambion^®^ WT Expression Kit) (Ambion, TX, USA). The obtained cDNA was used for biotin labeling and fragmentation using AffymetrixGeneChip^®^ WT Terminal Labeling and Hybridization kit (Affymetrix, Santa Clara, CA, USA). Biotin-labeled fragments of cDNA (5.5 µg) were hybridized to Affymetrix^®^ Rat Gene 2.1 ST Array Strip (48°C/20 h). Then, microarrays were washed and stained according to the technical protocol, using Affymetrix GeneAtlas Fuidics Station. The array strips were scanned employing Imaging Station of GeneAtlas System. The preliminary analysis of the scanned chips was performed using AffymetrixGeneAtlasTM Operating Software. The quality of gene expression data was checked according to quality control criteria provided by the software. Obtained CEL files were imported into downstream data analysis.

All analyzes were performed using BioConductor software, based on the statistical R programming language. For background correction, normalization, and summation of raw data, the Robust Multiarray Averaging algorithm implemented in “affy” package of BioConductor was applied (28). Biological annotation was taken from BioConductor “oligo” package where annotated data frame object was merged with normalized data set, leading to a complete gene data table (29).

The selection criteria of a significantly changed gene expression were based on expression fold difference higher than abs. 2 and adjusted *p* value <0.05. The result of such a selection was presented as volcano plots, where total number of up- and downregulated genes has been shown.

Data files were also deposited in the Gene Expression Omnibus (GEO) repository at the National Center for Biotechnology Information (http://www.ncbi.nlm.nih.gov/geo/) under the GEO accession number GEO: GSE93726.

### Assignment of the Genes Regulated by Sex Hormones to Relevant Gene Ontology Terms

Sets of female and male genes regulated by sex hormones were also subjected to functional annotation clusterization using DAVID web-based bioinformatics tools (Database for Annotation, Visualization, and Integrated Discovery) (30). Gene symbols for up- or downregulated genes from each of the compared groups were loaded to DAVID by “RDAVIDWebService” BioConductor package (31). Functional annotation charts generated by DAVID with overrepresented gene annotations in gene ontology biological process database (BO BP FAT) were shown as box plot. Interested gene sets from DAVID functional annotation charts were also subjected to hierarchical clusterization algorithm and presented as a heatmaps. Such analyses were carried out separately for male and female data sets. Arbitrary signal intensities from selected genes were represented by colors (green—higher; red—lower expression value). Log2 signal intensity values for any single gene were resized to Row *Z*-score scale (from −1.5, the lowest expression, to +1.5, the highest expression for single gene). Additionally, gene names and calculated fold values were also shown.

### Gene Set Enrichment Analysis (GSEA)

Gene Set Enrichment Analysis is a computational method used for testing *a priori* defined gene sets (GO terms, pathways) for association with one of the two compared biological groups. The method uses Kolmogorov–Smirnov (K-S) statistical test for identification of significantly enriched or depleted groups of genes (32). GSEA analysis has been conducted using GSEA Java Desktop Application from Broad Institute (http://software.broadinstitute.org/gsea/index.jsp). Normalized data from all of genes were transformed to an appropriate format and imported to application. Then, a predefined gene sets database named Hallmark was selected from Molecular Signatures Database (33). Genes belonging to the selected set were ranked according to the difference in their expression level using signal-to-noise ratio with 1,000 times permutation. By walking down the ranked list of genes, the enrichment score (ES) was calculated for each selected gene set. It was done by sum statistic when a gene was present in the gene set and decreasing it when it was not (34). ESs were normalized by their gene set size, and false positive finding were corrected by FDR.

### QPCR

QPCR was performed by means of the Lightcycler 2.0 instrument (ROCHE) with the 4.05 software version. SYBR green detection system was applied as described earlier (19, 25–27, 35–37). Every 20 μl reaction mixtures contains 2 μl template cDNA (standard or control), 0.5 μM of specific primer, and a previously determined optimum MgCl_2_ concentration (3.5 μM for each reaction). LightCyclerFastStart DNA Master SYBR Green I mix (ROCHE) was used. The real-time PCR program included 10 min denaturation step to activate the Taq DNA Polymerase, followed by a three-step amplification program: denaturation at 95°C for 10 s, annealing at 56°C for 5 s, and extension at 72°C for 10 s. Specificity of reaction products was checked by determination of melting points (0.1°C/s transition rate). Expression of studied genes was related to B2m, only in case of Hcrtr2 to PBGD.

The primers used were designed by Primer 3 software (Whitehead Institute for Biomedical Research, Cambridge, MA, USA) (Table 1). They were purchased from the Laboratory of DNA Sequencing and Oligonucleotide Synthesis, Institute of Biochemistry and Biophysics, Polish Academy of Sciences, Warsaw.

**Table 1:** **Primers used for QPCR validation of selected genes**.

cDNA	Genbank accession number	Primer	Primer sequence (5′–3′)	Position	PCR product size (bp)
Hcrtr2	NM_013074.1	S	GGCTTATCTCCAAATATTCCG	782–802	68
		A	CTCTGAACCACAGAAGAAGT	831–849	
AR	NM_012502.1	S	ATCATCTCTGTGCAAGTGCCC	3,634–3,654	162
		A	CCCATGCCAGAGAAGTAGTG	3,776–3,795	
ERa	NM_012689.1	S	ATGATGGGCTTATTGACCAAC	1,248–1,268	137
		A	AGGATCTCCAACCAGGCACA	1,365–1,384	
ERb	NM_012754.1	S	TCTGTGTGAAGGCCATGATC	1,474–1,493	237
		A	GCAGATGTTCCATGCCCTTG	1,691–1,710	
ERRa	NM_001008511.2	S	CTCTCTACCCAAACGCCTCT	334–353	234
		A	CGCACACCCTCCTTGAGCAT	548–567	
Mc2r	NM_001100491.1	S	GGACAAGGGGGGAGGCAGA	110–118	201
		A	TGGCACAACTACATCAGGAC	281–300	
Hmgcr	NM_013134.2	S	GACGCAACCTCTACATCCG	1,966–1,984	142
		A	TAGTTACCACTGACCGCCAGA	2,087–2,107	
B2m	NM_012512.2	S	CTTGCAGAGTTAAACACGTCA	316–336	70
		A	CTTGATTACATGTCTCGGTC	366–385	
PBGD	NM_013168.2	S	GAAAGACCCTGGAAACCTT	522–540	148
		A	AGCTCATCCAGCTTCCGTA	651–669	

*Gene symbol, gene names, Genebank accession numbers, oligonucleotide sequences for sense and antisense primers, their position, and product size are shown*.

### Statistical Analysis

The applied statistical analyses of gene expression are parts of the softwares used. The RT-QPCR data, on the other hand, are expressed as the mean ± SE, and the statistical significance of the differences between the compared groups was estimated using the Student’s *t*-test.

## Results

To study the modulatory effects of sex hormones on adrenal gland transcriptome profile, adult female and male rats were gonadectomized and replaced with estradiol or testosterone. The efficacy of the treatments has been demonstrated by seminal vesicles and uterine weights. As expected, weight of the examined organs decreased significantly after removal of gonads, whereas the weight of seminal vesicles and uteri were increased in the groups replaced with sex hormone [uterine weight (g): sham operated 0.42 ± 0.03, OVX 0.06 ± 0.01, OVX + estradiol replaced 0.37 ± 0.02; seminal vesicle weights: sham operated 0.41 ± 0.03, ORX 0.09 ± 0.01, ORX + testosterone replaced 0.85 ± 0.03; in each group *n* = 6; mean ± SE].

In these experimental conditions, ORX increases serum corticosterone concentrations, an effect reversed by testosterone replacement (Figure S1 in Supplementary Material). In the female, on the other hand, ovariectomy reduces serum corticosterone levels, while estradiol replacement restores corticosterone levels to the control values. In ORX rats, serum levels of the total cholesterol, LDL, and HDL were elevated, an effect reversed by testosterone replacement (Figure S2 in Supplementary Material). In OVX rats, serum levels of the total cholesterol, LDL, and HDL were higher and of triglyceride lower than in sham-operated animals. Estradiol supplementation restores these changes to the normal values.

**Figure 1:**
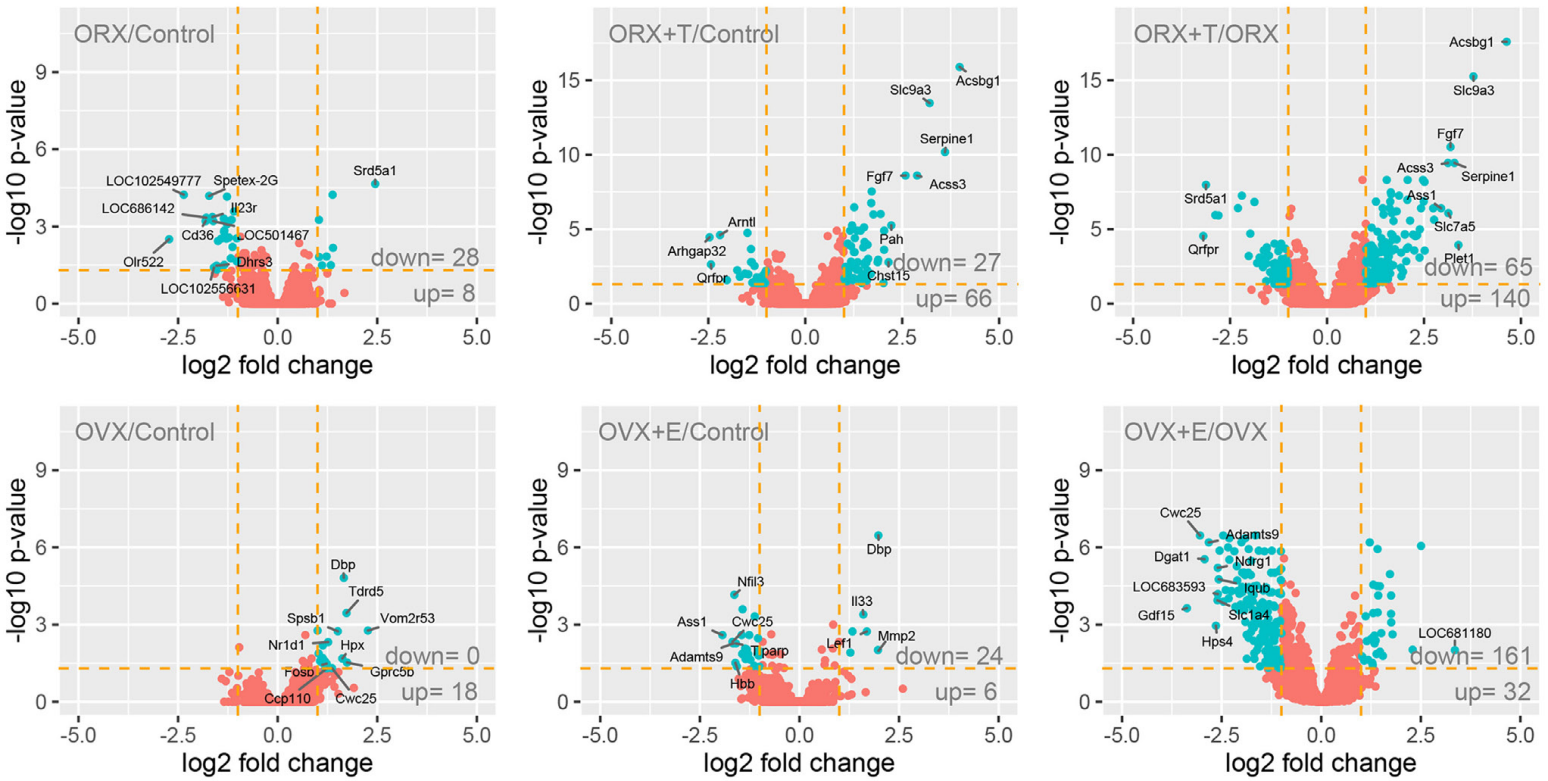
**Volcano plots showing the total gene expression profiles of adrenals of adult male and female control, gonadectomized and gonadal hormone replaced rats**. Each dot represents mean expression (*n* = 5) of a single gene obtained from microarray normalized dataset. Orange dotted cutoff lines were established using the following parameters: fold > |2| and adjusted *p* value <0.05. Genes above cutoff are considered as differentially expressed and shown as blue dots. Total numbers of differentially expressed genes are written in the right bottom corner of the graph. Ten of the most regulated genes are described by their gene symbols.

Adrenals obtained from five rats from each group were used for whole transcriptome analysis using Afymetrix microarray method, where expression of approximately 30,000 genes was examined. General gene expression profiles resulting from the comparison of male and female controls with appropriate experimental groups are shown as volcano plots (Figure 1). The selection criteria of a significantly changed gene expression level was based on expression fold difference higher than abs. 2 and adjusted *p* value <0.05. Considering these assumptions, ORX vs. control male adrenal transcription profile comparisons revealed that 36 genes were expressed differentially (28 down- and 8 upregulated genes). In the next compared groups: ORX + T vs. control males, we revealed 93 differentially expressed genes (27 down- and 66 upregulated). The last comparison of ORX + T vs. ORX groups revealed 205 differentially expressed genes (65 down- and 140 upregulated). Similar comparisons performed between OVX vs. control females, OVX + E vs. control females and OVX + E vs. OVX revealed that 18 (0 down- and 18 upregulated), 30 (24 down- and 6 upregulated), and 193 (161 down- and 32 upregulated) genes are differentially expressed, respectively.

**Figure 2:**
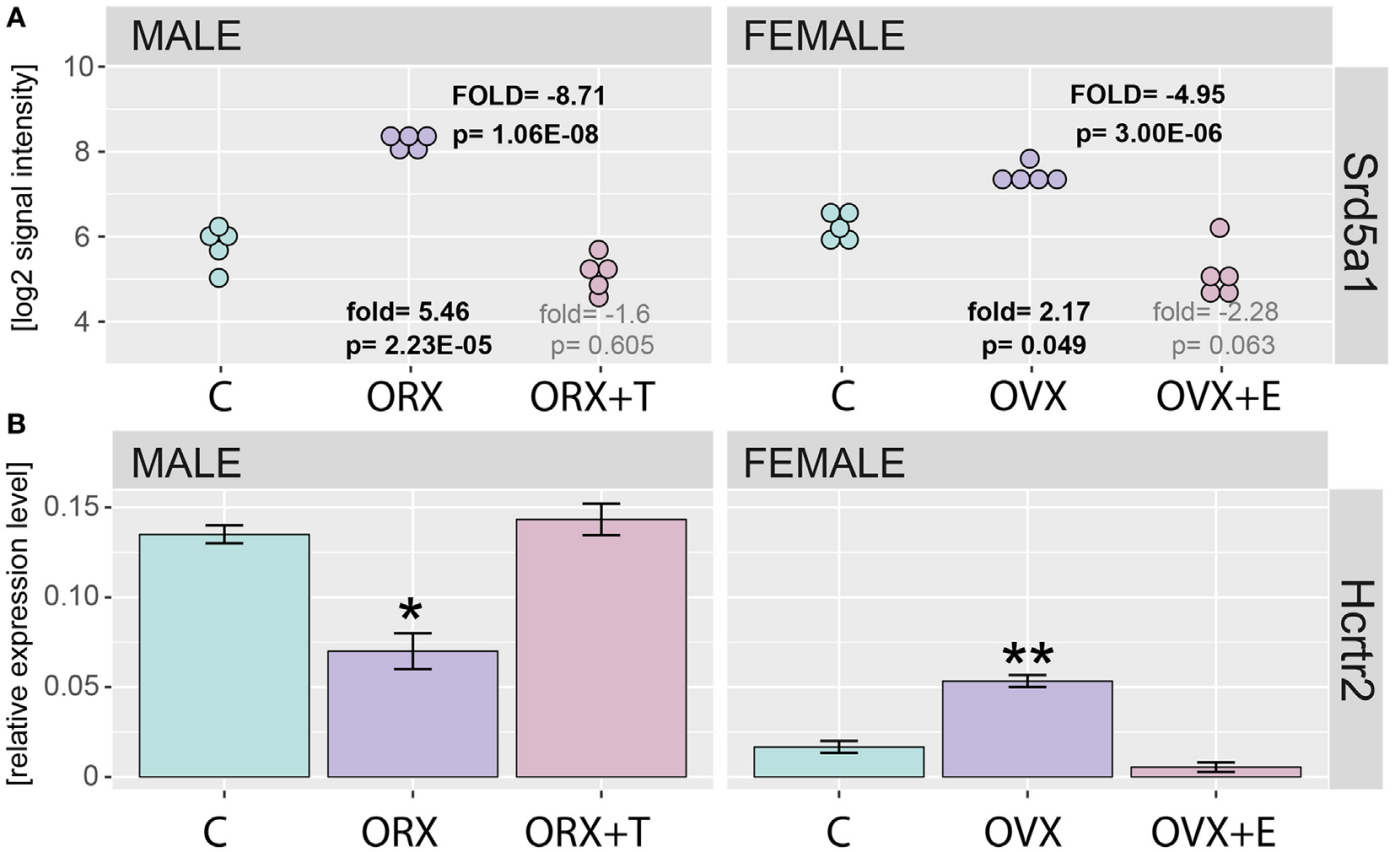
**Expression of Srd5a1 and Hcrtr2 genes in applied experimental model**. **(A)** Expression values were extracted from microarray normalized data set and presented as log2 signal intensity scale. Fold and *p* values were calculated in relation to appropriate control groups (showed on bottom) or between gonadectomized [orchiectomy (ORX) or ovariectomy (OVX)] and gonadectomized + sex hormone replaced (ORX + T, OVX + E) groups (showed on the top “FOLD” in capitals). In each group, *n* = 5. *p* Value obtained from moderated *t* test with FDR correction (incorporated in the Limma package of the software). **(B)** QPCR assay of Hcrtr2 genes in adrenals of adult male and female control, gonadectomized and gonadal hormone replaced rats. Bars represent means ± SEM (*n* = 5). HPRT gene was used as reference for data normalization. Statistically significant differences in relation to control group (Student’s *t*-test): *p < 0.05; **p < 0.02; ***p < 0.01; ****p < 0.001.

Correctness of presented study has been verified by detailed analysis of steroid-5-alpha-reductase alpha polypeptide 1 (Srd5a1) and hypocretin (orexin) receptor 2 (Hcrtr2) gene expression. Both, studied enzyme and receptor genes were earlier described as regulated by sex hormones. Profile of Srd5a1 gene expression was extracted from normalized microarray data set and presented on Figure 2A. Expression of Srd5a1 was significantly increased after gonadectomy (fold = 5.46, *p* = 2.23E-05 for male, and fold = 2.17, *p* = 0.049 for female, in relation to appropriate control groups) and reversed by sex hormone replacement (fold = −1.6, *p* = 0.605 for male and fold = −2.28, *p* = 0.063 for female, in relation to control groups). As revealed by QPCR, also expression of Hcrtr2 is notably decreased in ORX and reversed by testosterone. On the contrary, in OVX rats expression of Hcrtr2 was elevated and again this effect was reversed by estradiol replacement (Figure 2B).

**Figure 3:**
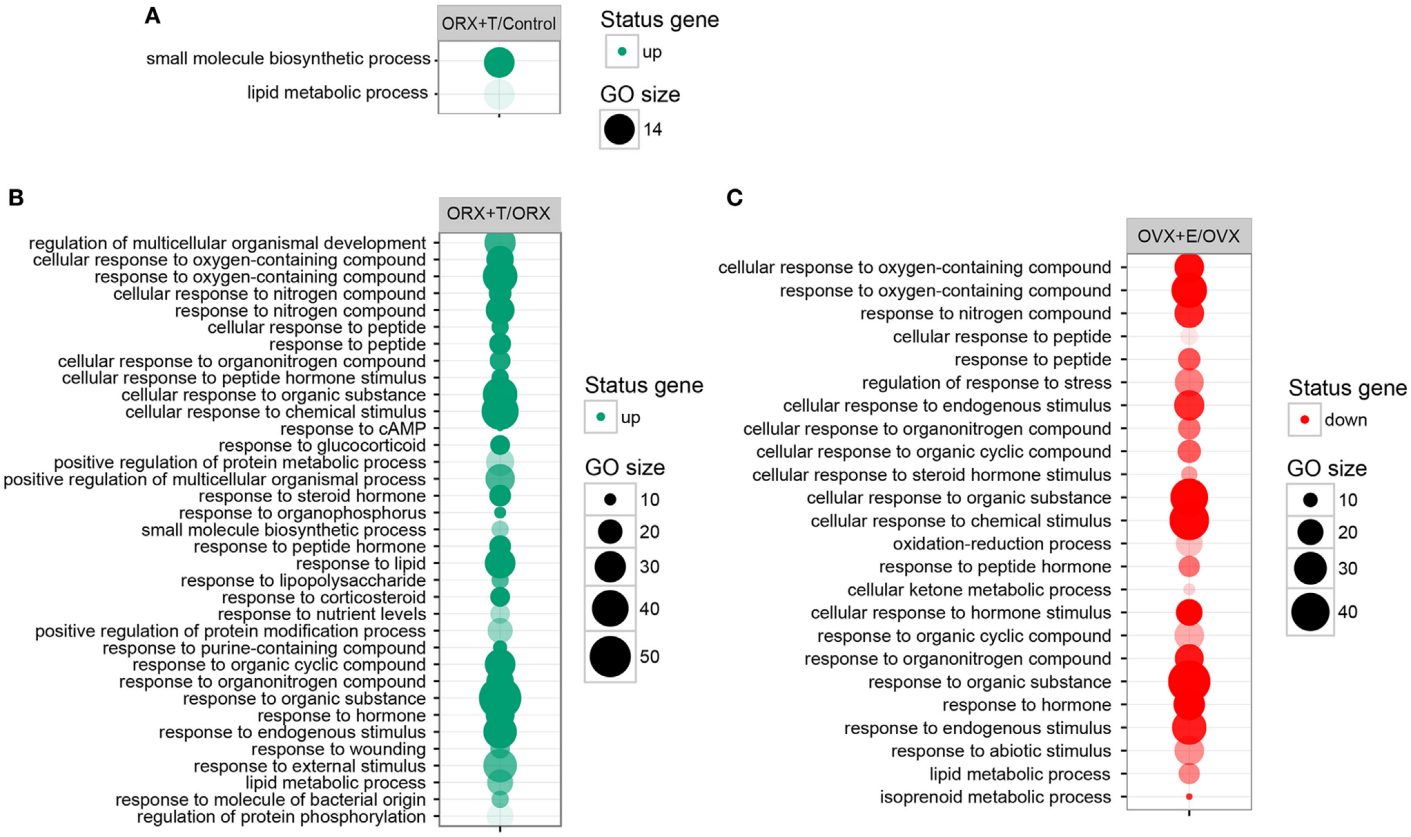
**Bubble plot of overrepresented gene set in DAVID GEOTERM BP FAT annotations database obtained from comparisons of expression profiles between: (A) male control/orchiectomy (ORX) + T, (B) ORX/ORX + T, (C) ovariectomy (OVX)/OVX + E**. The graphs show only the GO groups above the established cutoff criteria (*p* with correction <0.01, minimal number of genes per group >5). The size of each bubble reflects the number of differentially expressed genes, assigned to the GO BP terms. Transparency of the bubbles displays *p* value (more transparent—closer to the border of *p* = 0.01).

**Figure 4:**
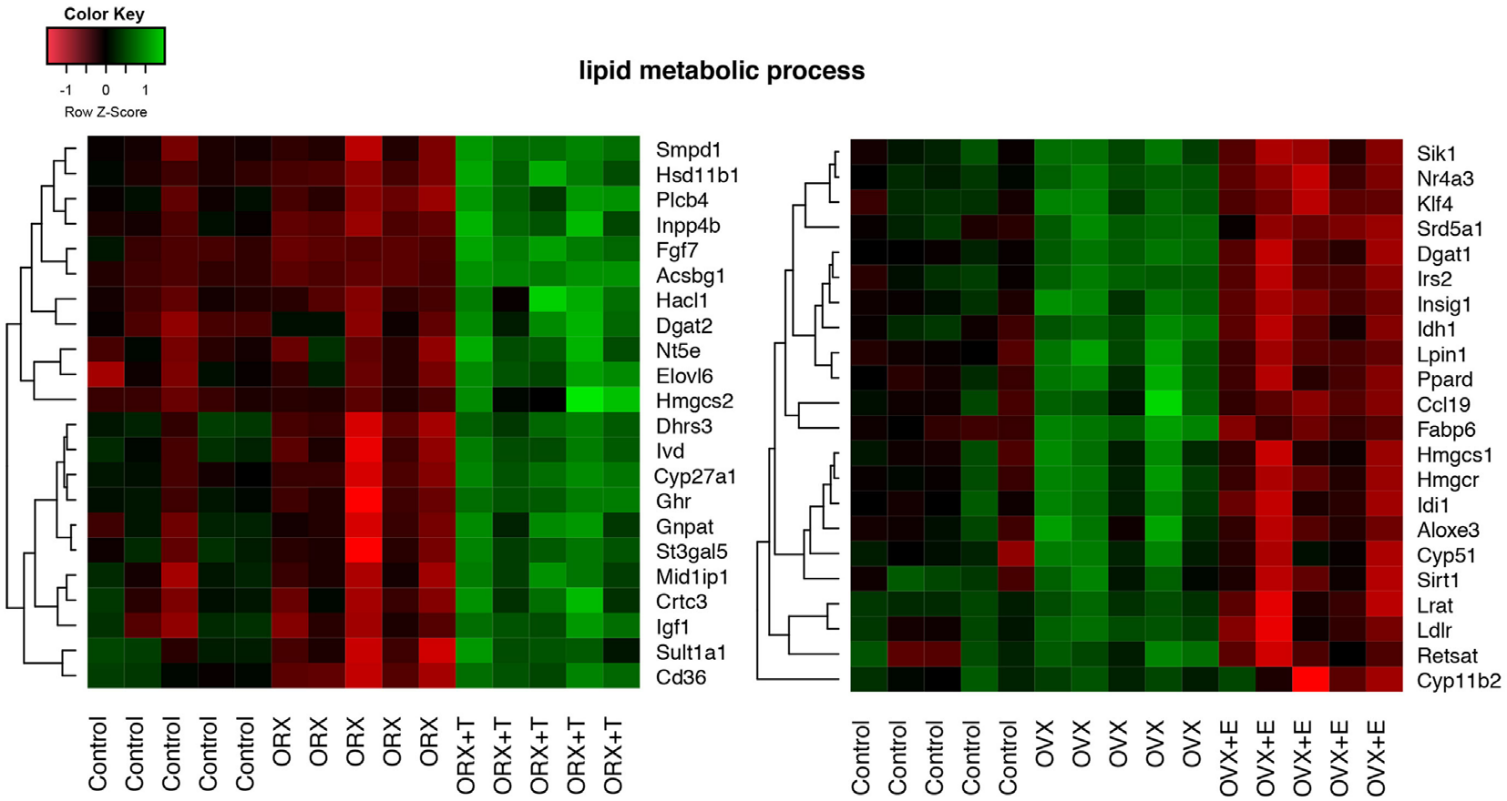
**Heat map graph of male and female adrenal gland genes regulated by sex hormones and belonging to “lipid metabolic process” GO BP term**. Arbitrary signal intensity acquired from the microarray analysis is represented by colors (green—higher; red—lower expression). Log2 signal intensity values for any single gene were resized to Row *Z*-score scale (from −1.5, the lowest expression, to +1.5, the highest expression for single gene). Presented genes are described by their gene symbols. Genes were selected from David GO terms, obtained by comparison of orchiectomy (ORX)/ORX + T and ovariectomy (OVX)/OVX + E. For better data understanding, the control groups have also been shown; however, they were not included into comparisons.

Differentially expressed gene sets were subsequently used for screening of their overrepresentation in some particular gene ontological groups. For this reason, gene symbols from all previously mentioned comparisons were loaded to DAVID searching system. Due to the small number of differentially expressed genes in ORX vs. control, OVX vs. control, and OVX + E vs. control, we did not obtain statistically significant GO groups in the abovementioned comparisons. Comparison between ORX + T vs. control males revealed two GO terms formed by differentially expressed genes: “small molecule biosynthetic process” and “lipid metabolic process” (Figure 3A). Genes from these GO terms were upregulated in ORX + T group in relation to male control. In the next comparison, 35 GO terms were formed from genes differentially expressed in ORX + T vs. ORX groups. Some of obtained GO groups have a general meaning, e.g., “response to organic substance,” “response to chemical stimulus” but other seems to be more related to adrenal physiology, i.e., “response to steroid hormone” or “lipid metabolic process.” All of presented GO terms were upregulated in ORX + T group in relation to ORX (Figure 3B). The last presented GO term graph demonstrates OVX + E vs. OVX groups comparisons, which delivered 24 GO terms. It is worth to notice that genes formed presented GO terms are downregulated in OVX + E vs. OVX group (Figure 3C). In this comparison, “lipid metabolic process” term was also statistically significant, and for this reason, genes belonging to this process in ORX + T vs. ORX as well as OVX + E vs. OVX were selected for hierarchical clusterization and presented as heatmap on Figure 4. To obtain the maps, 22 genes were selected from the “lipid metabolic process” in both male and female comparisons. All genes from male adrenals belonging to the “lipid metabolic process” are characterized by the highest expression levels in ORX + T group (green color) and the lowest in ORX group (red color). Expression of female genes has opposite profile. The highest gene expression levels are in OVX (green color) group, while the lowest in OVX + E (red color).

**Figure 5:**
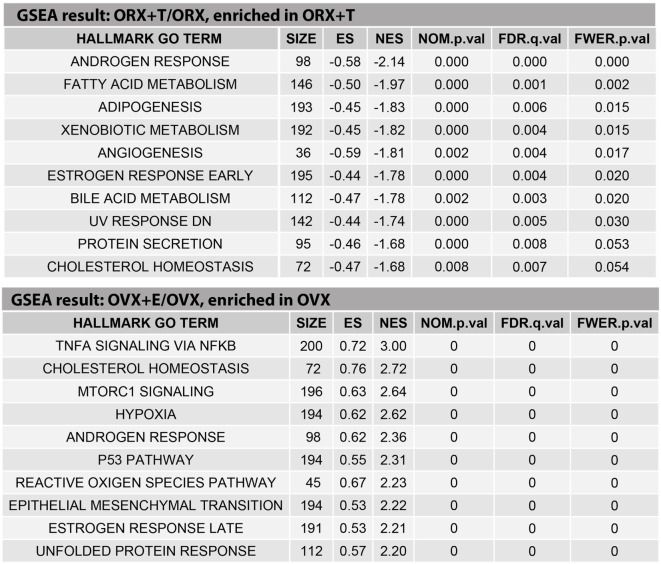
**Ten of the highest enriched HALLMARK GO TERMS obtained from gene set enrichment analysis (GSEA) between orchiectomy (ORX)/ORX + T and ovariectomy (OVX)/OVX + E groups**. Size of the category (number of genes in the “TERM”), enrichment score (ES), normalized enrichment score (NES), *p* value (NOM.p.val), *q* value of FDR correction (FDR.q.val), and *p* corrected by FDR (FWER.p.val) are shown.

**Figure 6:**
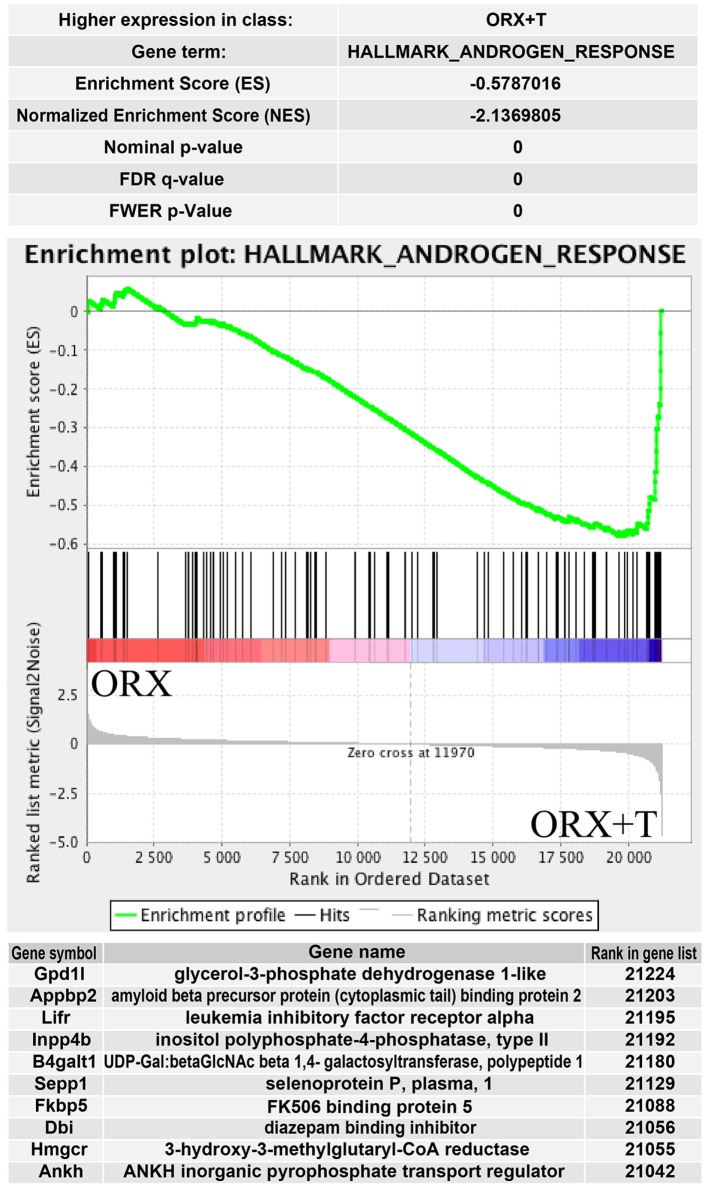
**Summary results of gene set enrichment (GSEA) in HALLMARK_ANDROGEN_RESPONSE term**. Enrichment analysis was performed between orchiectomy (ORX) and ORX + T groups of male adrenal glands. Normalized enrichment score (NES) as well as *p* values are shown. Middle plot presents rank of each gene belonging to androgen response term with enrichment score (ES) peak values. Ten of the top relevant genes for enrichment calculation are shown in table with their symbol, names, and rank in gene list.

**Figure 7:**
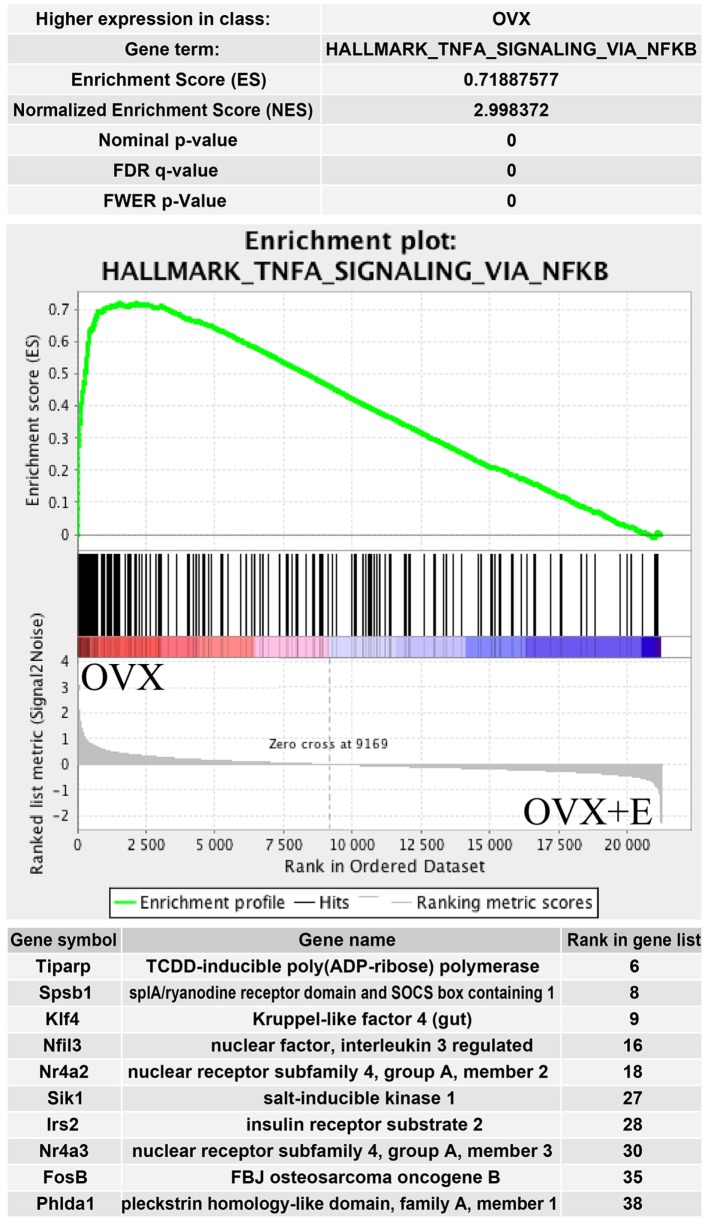
**Summary results of gene set enrichment (GSEA) in HALLMARK_TNFA_SIGNALING_VIA_NFKB term**. Enrichment analysis was performed between ovariectomy (OVX) and OVX + E groups of female adrenal glands. Normalized enrichment score (NES) as well as *p* values are shown. Middle plot presents rank of each gene belonging to Tnfa signaling via NFKB term with enrichment score (ES) peak values. Ten of the top relevant genes for enrichment calculation are shown in table with their symbol, names, and rank in gene list.

The above presented results were confirmed by another powerful bioinformatics tool that is the Gene Set Enrichment Analysis (GSEA). These analyzes were performed for: OXR/OXR + T and OVX/OVX + E experimental groups. After uploading of normalized expression level data from microarray to the software, we obtained a list of significantly represented terms from Hallmark database software. Ten of the most enrichment terms from each of the comparisons are presented on Figure 5. In accordance with DAVID and heatmap results, presented terms were enriched, it means that expressions of genes were higher in ORX + T (ORX + T/ORX) and OVX (OVX + E/OVX). The strongest enriched term in the comparison between ORX + T/ORX refers to “androgen response” while “TNFA signaling via NFKB” term was the strongest enriched in GSEA between OVX + E/OVX. Detailed results of those two terms are presented in Figures 6 and 7. List of the top 10 genes that strongly affect ES with their rank position are also shown.

**Figure 8:**
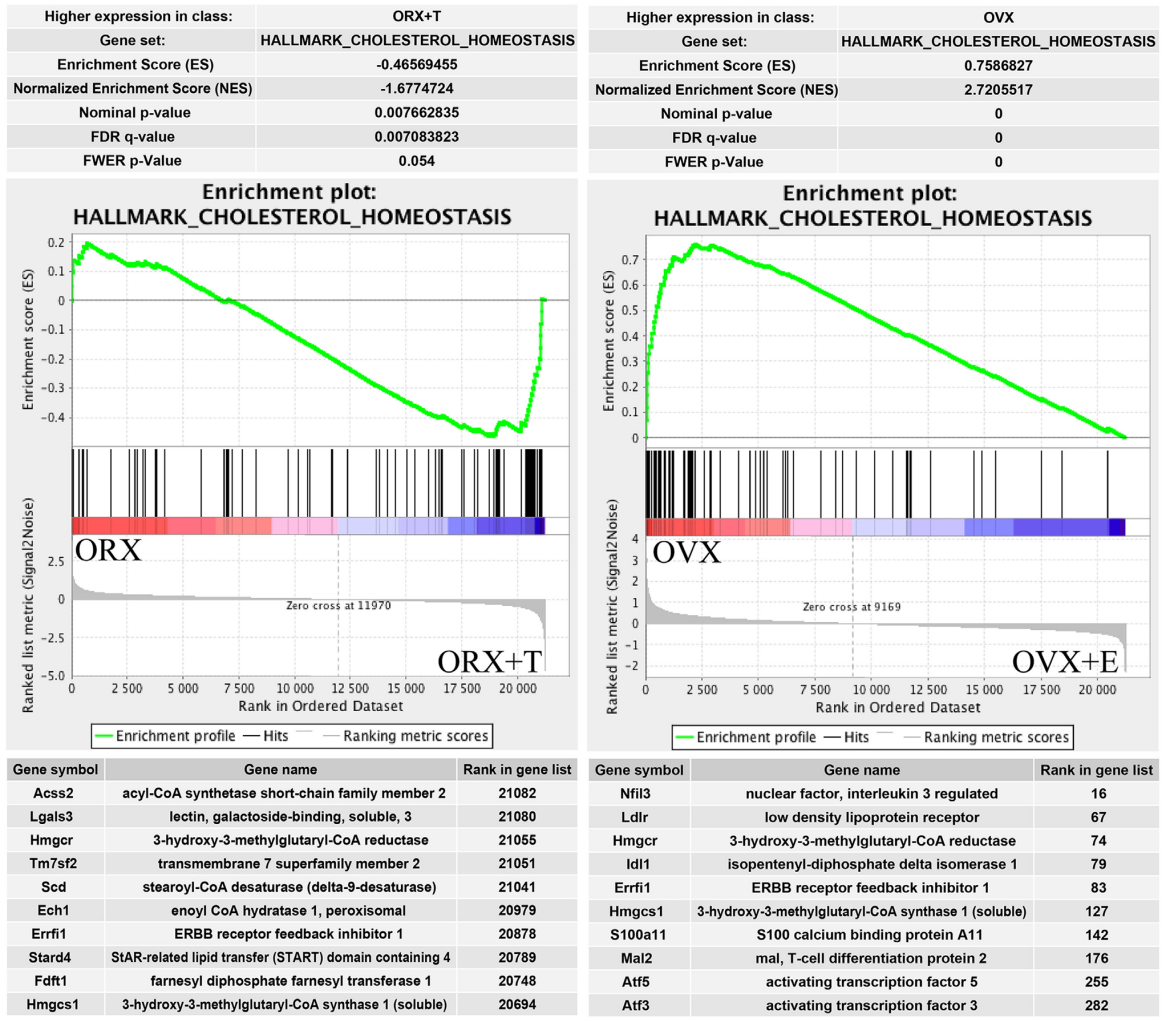
**Summary results of gene set enrichment (GSEA) in HALLMARK_CHOLESTEROL_HOMEOSTASIS term**. Enrichment analyses were done between gonadectomized and gonadectomized + sex hormone replaced groups, in both sexes. Normalized enrichment score (NES) as well as *p* values are shown. Middle plots present ranks of each gene belonging to cholesterol homeostasis term with enrichment score (ES) peak values. Ten of the top relevant genes for enrichment calculation from each sexes are shown in table with their symbol, names, and rank position.

One of the top enrichment term concerns the process strictly connected with adrenocortical steroidogenesis named “cholesterol homeostasis.” Results of GSEA are presented in Figure 8. Genes belonging to “cholesterol homeostasis” hallmark term are highly enriched in ORX + T group in relation to ORX group. Opposite results were obtained in OVX/OVX + E comparison, where gene belonging to “cholesterol homeostasis” are enriched in OVX group, it means that the expressions are higher in OVX in relation to OVX + E. Obtained results from GSEA are in commitment with previously presented DAVID GO analysis. List of the top 10 genes with their rank position are also shown in these figures.

**Figure 9:**
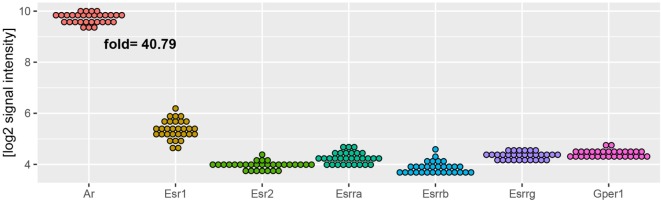
**Expression of androgen (Ar) and estrogen (Esr1, Esr2, Esrra, Esrrb, Esrrg, Gper) receptors in adrenal glands of studied rats (male and female control, gonadectomized and gonadal hormone replaced rats)**. Data obtained from Affymetrix^®^ Rat Gene 2.1 ST Array. From microarray data sets, expression values of analyzed genes were extracted. Each dot on graph corresponds to single log2-transformed value of signal intensity from individual sample. Fold expression change was calculated from mean of Ar receptor gene and mean of all estrogen receptors.

**Figure 10:**
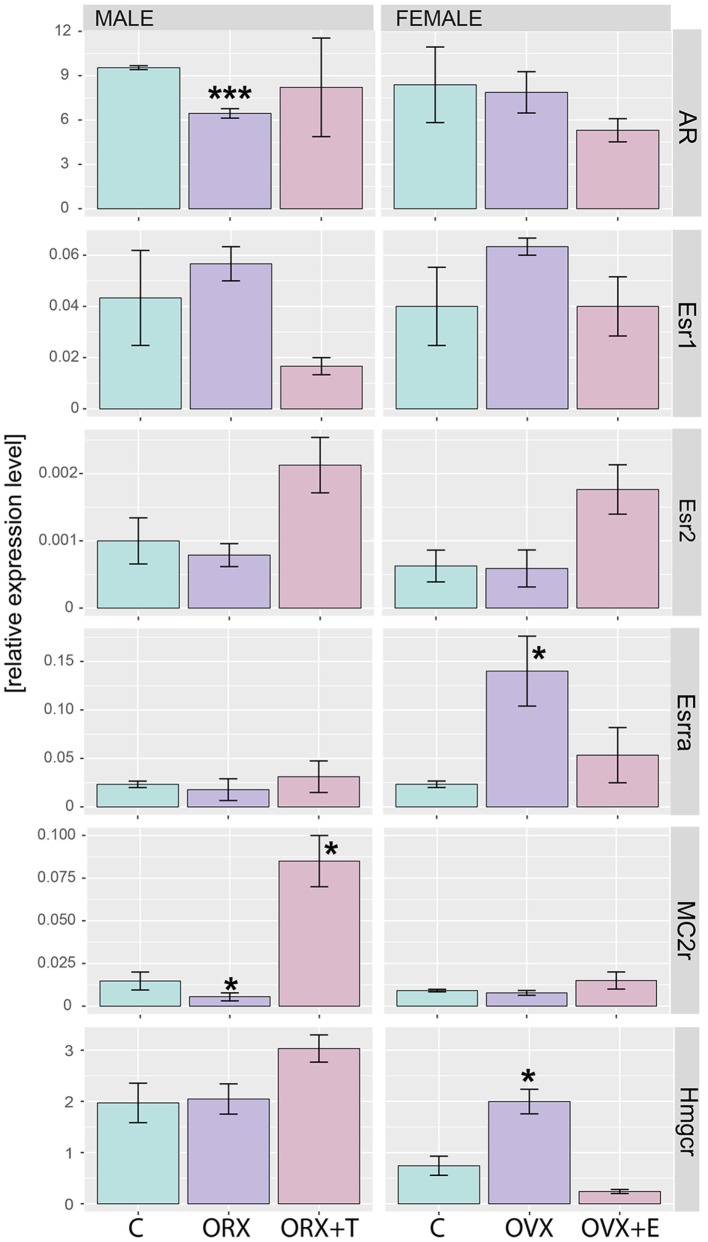
**QPCR assay of AR, Esr1, Esr2, Esrra, MC2r, and Hmgcr genes in adrenals of adult male and female control, gonadectomized and gonadal hormone replaced rats**. Bars represent mean ± SEM (*n* = 3). All samples were amplified in triplicate, and HPRT gene was used as reference for data normalization. Statistically significant differences in relation to control group: *p < 0.05; ***p < 0.01.

Regardless of experimental group, AR mRNA (androgen receptor) levels in rat adrenal are over 40 times higher than levels of estrogen receptors (Figure 9). As demonstrated by means of QPCR, expression levels of AR mRNA were not changed after OVX and OVX + E in relation to adrenals of control female rats. However, expression of AR was decreased after ORX and restored to control value after testosterone replacement. In both sexes, gonadectomy and sex hormone replacement did not change Esr1 and Esr2 gene expression. Expression of Esrra gene, on the other hand, was significantly elevated after OVX and restored to control level after estrogen replacement. Expression of Esrra in male adrenals was not changed in any experimental conditions. Expression of Mc2r was not significantly regulated in any experimental conditions in female adrenals, whereas it was lowered in gonadectomized males and elevated in testosterone-replaced animals. It is interesting to note that Hmgcr gene expression in the adrenal gland of male rats is significantly higher than in females. We also revealed that expression levels of this gene were affected neither by ORX nor testosterone replacement. On the other hand, ovariectomy resulted in a notable increase in Hmgcr gene expression levels, an effect reversed by estradiol replacement (Figure 10).

## Discussion

For many years, it is known that the multidirectional actions of androgen and estrogen lead to obvious sex differences in the structure and function of the adrenal cortex. As a result, in the adult female rat, adrenal cortex is larger and secretes more corticosterone than that of males (references in Section “Introduction”). It is commonly assumed that this effect depends on the stimulatory effect of estrogen on the HPA axis, while androgens have the opposite effect.

As revealed by global gene profiling method, distinct sex differences in the rat adrenal gland are accompanied by notable differences in expression levels of various groups of genes (19). We demonstrated that expression levels of genes involved in corticosteroid synthesis are higher and those regulating basal cell functions lower in adrenals of adult female rats. However, the control of adrenocortical gene expression by sex hormones is totally unknown. Regarding this, only one study presents data on global gene profiling in the adrenals of female intact and testosterone—administered mice (20). However, this experimental model is not a physiological one. To get information about the regulation by testosterone and estradiol of adrenal genes in male and female rats, we used the classic “remove and replace” experiments. As evidenced by changes in uterine and seminal vesicles weights, expected, gonadectomy-induced drop in the weight of these tissues was reversed by replacement with appropriate sex hormone.

In light of the above presented discussion on sex differences in adrenals of rats, one would expect the stimulatory effect of estradiol and the opposite effect of testosterone on the glands. To our surprise, global gene profiling analyses performed on adrenals of studied rats delivered rather unexpected results. It appeared that testosterone replacement mainly upregulates expression of adrenal genes while estradiol effect is opposite, large group of genes was downregulated. Regarding these results, the correctness of our experiments we tried to confirm by the more detailed studies of genes whose expression in a rat adrenal gland is known as dependent on testosterone or estradiol.

One of them is steroid-5-alpha-reductase alpha polypeptide 1 (Srd5a1). Expression of this gene and activity of the coded enzyme are notably elevated in ORX and OVX rats, changes prevented by testosterone or estradiol replacement, respectively (5, 38–43). Our microarray data are consistent with these findings. The second studied gene is orexin receptor 2 (Hcrtr2). Higher expression of Hcrtr2 in adrenals of the male rat in relation to female one as well as stimulatory effects of testosterone and inhibitory action of estradiol on this gene are well documented (19, 44–46). And again, our QPCR data are consistent with the earlier reports. Thus, obtained in the experiments performed, data on expression of specific genes in adrenals of rats of both sexes are consistent with earlier reports. In view of these, results still remains open while the global profile of gene expression regulated by sex hormones in the rat adrenal gland suggests prevalent inhibitory effect of estradiol and stimulatory effect of testosterone.

To gain an insight into this problem, we performed numerous additional analyses of data delivered by Affymetrix microarray system. As demonstrated by Volcano plots, in adrenals of experimental rats, only small number of differentially expressed genes was found. Differentially expressed genes were further analyzed in order to get their representation in GO. This was accomplished by DAVID web-based bioinformatics tools. The highest numbers of GO terms were obtained in comparisons of ORX + T vs. ORX experimental groups (35 GO terms). In females, on the other hand, analogical comparison (OVX + E vs. OVX) delivered 24 GO terms. It is noteworthy to underline that testosterone replaced rat genes in obtained groups were upregulated, while those in estradiol-replaced ovariectomized animals were downregulated. One of these GO terms—“lipid metabolic process”—seems to be related to adrenal physiology. This term occurs in both, testosterone- or estradiol-replaced groups; however, in androgen-replaced rats, expression levels of genes included in this group are elevated, while in estrogen supplemented animals their expression is lowered.

Our results obtained by the DAVID web-based bioinformatic tools were confirmed by GSEA. GSEA revealed that in ORX + T group (in relation to ORX) genes belonging to the Hallmark GO terms “androgen response,” “fatty acid metabolism,” “adipogenesis,” and “cholesterol homeostasis” are notably enriched. Moreover, in OVX group (in relation to OVX + E), enriched genes are present, among others, within the following terms: “TNFA signaling via NFKB,” “cholesterol homeostasis,” and “androgen response.” These data suggest that in adrenals of gonadectomized rats both, testosterone or estradiol replacement, potently affects genes involved in lipid and cholesterol metabolism, as well as intracellular pathways responsible for androgen response. Further GSEA analysis revealed that within the hallmark term “cholesterol homeostasis,” in both, orchiectomized testosterone- and ovariectomized estradiol-replaced rats, among 10 genes that strongly affect ES Hmgcr is present.

There is some evidence that total lipid and cholesterol concentrations and contents in the rat adrenal gland are influenced by gonadal hormones. Lowered concentration of total lipid in adrenals of gonadectomized rats of both sexes was reported by Coleman et al. (47). Higher total lipid content, but not total lipid concentration in adrenals of female than male rats, has been reported by other group (48, 49). These studies demonstrated also higher concentrations and contents of total lipid in adrenals of orchiectomized testosterone replaced rats, while an opposite effects were observed in ovariectomized rats replaced with estradiol.

Although cholesterol is not a classical lipid, its physiological role in the adrenal cortex is often described in the aspects of the role of lipids. In contrast to lipids, numerous data are available on cholesterol and cholesterol esters in adrenals of intact male and female rats and on impact on them of sex hormones.

HMG-CoA reductase is the rate-limiting enzyme in cholesterol biosynthesis (50, 51). Administration of 17α-ethinyl-estradiol into aging male rats resulted in a notable stimulation of enzyme activity (52). Our results indicate that testosterone has no effect on the expression of Hmgcr gene; however, ovariectomy increases its expression, an effect reversed by estradiol replacement. Regarding these changes, it should be emphasized that HMG-CoA reductase is a highly regulated gene, among others by serum lipoprotein levels and intracellular cholesterol content (50, 53). Thus, it seems legitimate to suggest that observed in the present study changes in Hmgcr expression may be mediated by both, serum lipoprotein levels and adrenal cholesterol content. This assumption is supported by parallel changes in adrenal HMG-CoA reductase gene expression and serum HDL concentrations in studied female rats.

According to data reviewed in the earlier monographs, the majority of publications indicate a higher concentration of cholesterol (both, free or esterified) in the adrenal glands of adult female rats (5, 49). ORX increased cholesterol concentration in the gland, an effect reversed by testosterone replacement. Ovariectomy, on the other hand, either did not change or lowered the total cholesterol concentration in the rat adrenal gland. There is general agreement, on the other hand, that estrogens lower cholesterol content in adrenals of ovariectomized rats.

Free cholesterol, required for steroid hormone synthesis, is liberated from cholesterol esters by the action of ACTH-sensitive sterol ester hydrolase. The specific activity of this enzyme in adrenal homogenates is notably higher in male than female glands (54). Both, ORX and testosterone replacement had any effect on adrenal sterol ester hydrolase. Ovariectomy, on the other hand, resulted in an increase in activity of the enzyme, an effect reversed by estradiol replacement.

Thus, earlier data on the effects of testosterone and estradiol on adrenal lipids and enzymes involved in cholesterol metabolism in the rat are rather consistent with the transcriptome profile obtained in the respective experimental groups.

Also genes forming the hallmark GO term “androgen response” in rat adrenals are highly regulated by both, testosterone or estradiol replacement. In an aspect of this term in the present study, we analyzed the expression of the androgen receptor. Regardless of experimental group, AR mRNA levels in rat adrenal are over 40 times higher than levels of estrogen receptors, differences observed also by others (27, 55–59). We observed that expression of AR was decreased after ORX and restored to control value after testosterone replacement while no changes were observed after OVX and OVX + E. Our results are also consistent with the data on the sheep (60). Thus, our data did not confirm earlier reports on the effects of gonadectomy and sex hormone replacement on the levels of androgen- or estrogen-binding sites in the rat adrenals (56–58). Of estrogen receptor isoforms studied, only Esrra (estrogen related receptor alpha, also known as NR3B1) is overexpressed in ovariectomized rats and restored to normal level by estradiol replacement.

In adrenals of female rats, hallmark GO term “TNFA signaling via NFKB” is the first term delivered by GSEA, with the size 200 (number of genes). Such a huge enrichment is observed in OVX rats, in relation to OVX estradiol-replaced rats. TNFA is a proinflammatory cytokine and TNF-induced NFKB activation induces transcription and expression of genes involved in various biological processes, including regulation of cell proliferation, differentiation, apoptosis, and immune response. The final steps of these signaling pathways are mediated through activation of nuclear transcription factors (61–63). Also other intracellular signaling pathways are affected by estradiol replacement, they predominantly downregulate respective genes. These findings may suggest inhibition of expression of numerous transcription factor genes in OVX + E female rats. Mechanism of such actions remains to be solved.

It is well documented that androgen and estrogen receptors are ubiquitously expressed in almost all tissues and organs. Therefore, *in vivo* experiments do not allow for precise identification of their action on the adrenal glands. Sex hormones exert a multidirectional effect on the function of the HPA axis, affecting primarily CRH and ACTH synthesis and/or secretion [for review, see Ref. (3–6, 64)]. Most of the experimental data based on gonadectomy and sex hormone replacement indicate that in the rat estrogens stimulate and androgens inhibit the HPA axis. However, sex hormones may also exert direct effects on the rat adrenal cortex. These effects may be mediated either via specific receptors, both nuclear and membrane (for example GPR30) or by interference with ACTH-sensitive intracellular pathways, particularly those associated with steroidogenesis (65–69). Important in the physiological control of HPA axis is also liver. This organ is critical for the regulation of CBG and lipoproteins and metabolizes circulating corticosteroids. As known, all these functions are regulated by sex hormones.

As demonstrated by means of QPCR, expression of Mc2r was not significantly regulated in any experimental conditions in female adrenals, whereas it was lowered in gonadectomized males and elevated in testosterone-replaced animals. In terms of gender differences, very contradictory data relate to gender differences in the content and secretion of ACTH by the rat pituitary gland and corticotrophin levels in blood serum are available [for review, see Malendowicz (5)]. The major part of these studies show a higher level of ACTH in the blood serum of rats treated with estrogen. Only scanty data, on the other hand, are available on expression of ACTH receptors in adrenals of male and female rats. Yoshimura et al. (70) have shown that expression levels of ACTH receptor mRNA in adrenals increased with age in the female rat, but not in the male. In these studies, ORX elevated the level of ACTH receptor, an effect reversed by testosterone replacement. In contrast, our experiments demonstrated that ORX resulted in lowering of Mc2r gene expression and testosterone replacement notable increased expression of this gene. It is difficult to explain these discrepancies, most likely they are the result of different experimental models and methods applied for determining the expression of a discussed gene [male rats aged 14 days (period of temporal decrease of adrenal ACTH responsiveness) vs. adult animals; size and quality of applied primers]. Our results, however, are consistent with the data on the sheep. In this species, estradiol did not change adrenal Mc2r expression, while higher Mc2r expression was found in testosterone-treated rams (60).

In view of the above literature data for stimulating effects of estradiol and inhibitory of testosterone on the growth and function of the adrenal cortex of rat, obtained results of the analysis of transcriptome of sex hormone-replaced rats are surprising. Contrary to what we expected to find, it appeared that testosterone replacement resulted in a potent stimulation of expression of numerous adrenal genes, especially those involved in lipids and cholesterol metabolism. On the other hand, estradiol replacement resulted in a strong downregulation of numerous genes, especially those involved in regulation of TNFA signaling via NFKB pathways and on transcription factors synthesis. Obtained results may suggest that estradiol inhibits the expression of corepressor genes in the rat adrenal gland. It seems possible that this mechanism of action may lead to an increase in both the adrenal gland growth and secretory function. The physiological relevance of these findings await further research.

## Ethics Statement

This study was carried out in accordance with the recommendations of the “Directive 2010/63/EU of the European Parliament and of the Council of 22 September 2010 on the protection of animals used for scientific purposes.” as stated in the Polish law “Ustawa z dnia 15 stycznia 2015 r. o ochronie zwierzat wykorzystywanych do celów naukowych lub edukacyjnych.” The protocol was approved by the Local Ethics Committee for Animal Research (Poznan, Poland) (in Polish: Lokalna Komisja Etyczna ds. Doświadczeń na zwierzętach w Poznaniu), permission number: LKE—11/2015.

## Author Contributions

KJ invented and designed the experiments, performed the experiments, and performed microarrays; PC performed the experiments, QPCR; MS performed the experiments and performed QPCR; MT performed the experiments and performed QPCR; PM performed the experiments and performed QPCR; LM invented and designed the experiments, analyzed the data, and prepared the manuscript; MR invented and designed the experiments, analyzed the data, prepared graphical presentation, and prepared the manuscript.

## Conflict of Interest Statement

The authors declare that there is no conflict of interest that could be perceived as prejudicing the impartiality of the research reported.

## Supplementary Material

Figure S1 **Serum corticosterone concentrations in adult male and female control, gonadectomized and gonadal hormone replaced rats**. Bars represent mean ± SEM. Each circle represents an individual value. Statistically significant differences in relation to control or gonadectomized group: **p* < 0.05; ***p* < 0.02; ****p* < 0.01; *****p* < 0.001.

Figure S2 **Serum total cholesterol, LDL, HDL, and triglyceride concentrations in adult male and female control, gonadectomized and gonadal hormone-replaced rats**. Bars represent mean ± SEM. Each circle represents an individual value. Statistically significant differences in relation to control or gonadectomized group: **p* < 0.05; ***p* < 0.02; ****p* < 0.01; *****p* < 0.001.

Table S1 **List off all regulated genes in adrenals of adult male and female control, gonadectomized and gonadal hormone replaced rats**. Their fold change, *p* values, and their localization to chromosomes.

## References

[1] Bachmann R Handbuch der Mikroskopischen Anatomie des Menschen. Berlin, Göttingen, Heidelberg: Springer (1954).

[2] Kitay JI Effects of estrogen and androgen on the adrenal cortex of the rat. In: McKerns KW, editor. Functions of the Adrenal Cortex. Vol. 2 Amsterdam: North Holland Publishing Corpany (1968). p. 775–811.

[3] Kime D Vinson GP Major P Kilpatrick R Adrenal-gonad relationship. In: McKerns KW, editor. Functions of the Adrenal Cortex. Vol. 3 London: Academic Press (1979). p. 183–347.

[4] Handa RJ Weiser MJ. Gonadal steroid hormones and the hypothalamo-pituitary-adrenal axis. Front Neuroendocrinol (2014) 35:197–220. 10.1016/j.yfrne.2013.11.001 24246855 PMC5802971

[5] Malendowicz LK Cytophysiology of the Mammalian Adrenal Cortex as Related to Sex, Gonadectomy and Gonadal Hormones. Poznan: PTPN Press (1994).

[6] Goel N Workman JL Lee TT Innala L Viau V. Sex differences in the HPA axis. Compr Physiol (2014) 4:1121–55. 10.1002/cphy.c130054 24944032

[7] Green MR McCormick CM. Sex and stress steroids in adolescence: gonadal regulation of the hypothalamic-pituitary-adrenal axis in the rat. Gen Comp Endocrinol (2016) 234:110–6. 10.1016/j.ygcen.2016.02.004 26851306

[8] Gala RR Westphal U Corticosteroid-binding globulin in the rat: studies on the sex difference. Endocrinology (1965) 77:841–51. 10.1210/endo-77-5-841 5843904

[9] Rosner W The functions of corticosteroid-binding globulin and sex hormone-binding globulin: recent advances. Endocr Rev (1990) 11:80–91. 10.1210/edrv-11-1-80 2180689

[10] Mattos GE Heinzmann JM Norkowski S Helbling JC Minni AM Moisan MP Corticosteroid-binding globulin contributes to the neuroendocrine phenotype of mice selected for extremes in stress reactivity. J Endocrinol (2013) 219:217–29. 10.1530/JOE-13-0255 24048966

[11] Meyer EJ Nenke MA Rankin W Lewis JG Torpy DJ. Corticosteroid-binding globulin: a review of basic and clinical advances. Horm Metab Res (2016) 48:359–71. 10.1055/s-0042-108071 27214312

[12] Herbst AL Yates FE Glenister DW Urquhart J Variations in hepatic inactivation of corticosterone with changes in food intake: an explanation of impaired corticosteroid metabolism following noxious stimuli. Endocrinology (1960) 67:222–38. 10.1210/endo-67-2-222 14401221

[13] Kitay JI Sex differences in adrenal cortical secretion in the rat. Endocrinology (1961) 68:818–24. 10.1210/endo-68-5-818 13756461

[14] Glenister DW Yates FE Sex difference in the rate of disappearance of corticosterone-4-C14 from plasma of intact rats: further evidence for the influence of hepatic Delta4-steroid hydrogenase activity on adrenal cortical function. Endocrinology (1961) 68:747–58. 10.1210/endo-68-5-747 13706020

[15] Seal US Doe RP Vertebrate distribution of corticosteroid-binding globulin and some endocrine effects on concentration. Steroids (1965) 41:827–41. 10.1016/0039-128X(65)90174-1 14325185

[16] Kitay JI Effects of estradiol on pituitary-adrenal function in male and female rats. Endocrinology (1963) 72:947–54. 10.1210/endo-72-6-947 14033176

[17] Labrie F Interactions hormonales et rôle de la transcortine dans l’ajustement de l’activité hypophysosurrénalienne [These Doctorate Sciences]. Quebec: Universite Laval (1967).

[18] Kamoun A Activité cortico-surrénale au cours de la gestation, de la lactation et du développement pre- et postnatal chez le rat. J Physiol (1970) 65:5–32. 5486838

[19] Trejter M Hochol A Tyczewska M Ziolkowska A Jopek K Szyszka M Sex-related gene expression profiles in the adrenal cortex in the mature rat: microarray analysis with emphasis on genes involved in steroidogenesis. Int J Mol Med (2015) 35:702–14. 10.3892/ijmm.2015.2064 25572386 PMC4314423

[20] El Wakil A Mari B Barhanin J Lalli E. Genomic analysis of sexual dimorphism of gene expression in the mouse adrenal gland. Horm Metab Res (2013) 45:870–3. 10.1055/s-0033-1349881 23921913

[21] Cagen LM Baer PG. Effects of gonadectomy and steroid treatment on renal prostaglandin 9-ketoreductase activity in the rat. Life Sci (1987) 40:95–100. 10.1016/0024-3205(87)90257-8 3796215

[22] Longhurst PA Kauer J Leggett RE Levin RM. The influence of ovariectomy and estradiol replacement on urinary bladder function in rats. J Urol (1992) 148:915–9. 1512859 10.1016/s0022-5347(17)36777-0

[23] Udagawa K Ogawa T Watanabe T Tamura Y Kita K Kubota Y. Testosterone administration promotes regeneration of chemically impaired spermatogenesis in rats. Int J Urol (2006) 13:1103–8. 10.1111/j.1442-2042.2006.01484.x 16903937

[24] Schulte-Beerbühl M Nieschlag E Comparison of testosterone, dihydrotestosterone, luteinizing hormone, and follicle-stimulating hormone in serum after injection of testosterone enanthate of testosterone cypionate. Fertil Steril (1980) 33:201–3. 10.1016/S0015-0282(16)44543-7 7353699

[25] Tyczewska M Rucinski M Trejter M Ziolkowska A Szyszka M Malendowicz LK Angiogenesis in the course of enucleation-induced adrenal regeneration – expression of selected genes and proteins involved in development of capillaries. Peptides (2012) 38:404–13. 10.1016/j.peptides.2012.09.025 23041583

[26] Tyczewska M Rucinski M Ziolkowska A Szyszka M Trejter M Hochol-Molenda A Enucleation-induced rat adrenal gland regeneration: expression profile of selected genes involved in control of adrenocortical cell proliferation. Int J Endocrinol (2014) 2014:130359. 10.1155/2014/130359 25431590 PMC4241258

[27] Trejter M Jopek K Celichowski P Tyczewska M Malendowicz LK Rucinski M. Expression of estrogen, estrogen related and androgen receptors in adrenal cortex of intact adult male and female rats. Folia Histochem Cytobiol (2015) 53:133–44. 10.5603/FHC.a2015.0012 26122962

[28] Gautier L Cope L Bolstad BM Irizarry RA affy – analysis of Affymetrix GeneChip data at the probe level. Bioinformatics (2004) 20:307–15. 10.1093/bioinformatics/btg405 14960456

[29] Carvalho BS Irizarry RA. A framework for oligonucleotide microarray preprocessing. Bioinformatics (2010) 26:2363–7. 10.1093/bioinformatics/btq431 20688976 PMC2944196

[30] Huang da W Sherman BT Lempicki RA. Systematic and integrative analysis of large gene lists using DAVID bioinformatics resources. Nat Protoc (2009) 4:44–57. 10.1038/nprot.2008.211 19131956

[31] Fresno C Fernandez EA. RDAVIDWebService: a versatile R interface to DAVID. Bioinformatics (2013) 29:2810–1. 10.1093/bioinformatics/btt487 23958726

[32] Damian D Gorfine M Statistical concerns about the GSEA procedure. Nat Genet (2004) 36:663; author reply 663 10.1038/ng0704-663a 15226741

[33] Liberzon A Birger C Thorvaldsdottir H Ghandi M Mesirov JP Tamayo P. The Molecular Signatures Database (MSigDB) hallmark gene set collection. Cell Syst (2015) 1:417–25. 10.1016/j.cels.2015.12.004 26771021 PMC4707969

[34] Subramanian A Tamayo P Mootha VK Mukherjee S Ebert BL Gillette MA Gene set enrichment analysis: a knowledge-based approach for interpreting genome-wide expression profiles. Proc Natl Acad Sci U S A (2005) 102:15545–50. 10.1073/pnas.0506580102 16199517 PMC1239896

[35] Rucinski M Ziolkowska A Szyszka M Hochol A Malendowicz LK. Evidence suggesting that ghrelin *O*-acyl transferase inhibitor acts at the hypothalamus to inhibit hypothalamo-pituitary-adrenocortical axis function in the rat. Peptides (2012) 35:149–59. 10.1016/j.peptides.2012.04.007 22543218

[36] Trejter M Hochol A Tyczewska M Ziolkowska A Jopek K Szyszka M Visinin-like peptide 1 in adrenal gland of the rat. Gene expression and its hormonal control. Peptides (2015) 63:22–9. 10.1016/j.peptides.2014.10.017 25451331

[37] Szyszka M Paschke L Tyczewska M Rucinski M Grabowska P Malendowicz LK. Lack of expression of preproorexin and orexin receptors genes in human normal and prostate cancer cell lines. Folia Histochem Cytobiol (2015) 53:333–41. 10.5603/fhc.a2015.0035 26714447

[38] Kitay JI Coyne MD Swygert NH Adrenal 5 alpha-reductase activity in the rat: effects of gonadectomy: some cofactor and substrate requirements. Proc Soc Exp Biol Med (1971) 137:229–32. 10.3181/00379727-137-35550 4397241

[39] Colby HD Kitay JI Effects of gonadal hormones on adrenal steroid metabolism in vitro. Steroids (1972) 20:143–57. 10.1016/0039-128X(72)90066-9 5078258

[40] Malendowicz LK. Sex differences in adrenocortical structure and function. III. The effects of postpubertal gonadectomy and gonadal hormone replacement on adrenal cholesterol sidechain cleavage activity and on steroids biosynthesis by rat adrenal homogenates. Endokrinologie (1976) 67:26–35. 1244198

[41] Malendowicz LK Fichna P. Sex differences in adrenocortical structure and function. IV. Effects of postpubertal gonadectomy and sex hormones on adrenal steroid 5alpha-reductase activity in the rat. Endokrinologie (1978) 72:36–42. 688982

[42] Lephart ED Simpson ER Trzeciak WH. Rat adrenal 5 alpha-reductase mRNA content and enzyme activity are sex hormone dependent. J Mol Endocrinol (1991) 6:163–70. 10.1677/jme.0.0060163 2043243

[43] Yokoi H Tsuruo Y Ishimura K. Steroid 5alpha-reductase type 1 immunolocalized in the rat peripheral nervous system and paraganglia. Histochem J (1998) 30:731–9. 10.1023/A:1003482512567 9874000

[44] Johren O Neidert SJ Kummer M Dendorfer A Dominiak P. Prepro-orexin and orexin receptor mRNAs are differentially expressed in peripheral tissues of male and female rats. Endocrinology (2001) 142:3324–31. 10.1210/en.142.8.3324 11459774

[45] Johren O Bruggemann N Dendorfer A Dominiak P. Gonadal steroids differentially regulate the messenger ribonucleic acid expression of pituitary orexin type 1 receptors and adrenal orexin type 2 receptors. Endocrinology (2003) 144:1219–25. 10.1210/en.2002-0030 12639903

[46] Johren O Bruggemann N Dominiak P. Orexins (hypocretins) and adrenal function. Horm Metab Res (2004) 36:370–5. 10.1055/s-2004-814569 15241726

[47] Coleman RD Chen YM Alfinslater RB Cholesterol metabolism in gonadectomized rats. Circ Res (1958) 6:172–7. 10.1161/01.RES.6.2.172 13511672

[48] Malendowicz LK Mlynarczyk W. Sex differences in adrenocortical structure and function. X. Lipid and corticosterone in the rat adrenal as affected by gonadectomy and testosterone or estradiol replacement. Endokrinologie (1982) 79:292–300. 7128546

[49] Duda T Waliszewska A Trzeciak WH Malendowicz LK. Sex differences in adrenocortical structure and function. XX. The effects of gonadectomy and testosterone or estradiol replacement on cholesterol content and distribution in the gland. J Steroid Biochem (1985) 23:577–81. 10.1016/0022-4731(85)90007-X 3001407

[50] Goldstein JL Brown MS. Regulation of the mevalonate pathway. Nature (1990) 343:425–30. 10.1038/343425a0 1967820

[51] Brown MS Goldstein JL. Lipoprotein receptors: therapeutic implications. J Hypertens (1990) 8(Suppl):S33–5; discussion S35–6. 10.1097/00004872-199003001-00007 2185351

[52] Popplewell PY Azhar S. Effects of aging on cholesterol content and cholesterol-metabolizing enzymes in the rat adrenal gland. Endocrinology (1987) 121:64–73. 10.1210/endo-121-1-64 2885178

[53] Friesen JA Rodwell VW. The 3-hydroxy-3-methylglutaryl coenzyme-A (HMG-CoA) reductases. Genome Biol (2004) 5:248. 10.1186/gb-2004-5-11-248 15535874 PMC545772

[54] Trzeciak WH Malendowicz LK Sex differences in adrenocortical structure and function. VII. Adrenal sterol ester hydrolase activity in the rat and its dependence on gonadal hormones. Horm Metab Res (1981) 13:519–22. 10.1055/s-2007-1019321 7298023

[55] Muller RE Wotiz HH Estrogen-binding protein in mouse and rat adrenal glands. J Biol Chem (1978) 253:740–5. 621202

[56] Calandra RS Naess O Purvis K Attramadal A Djoseland O Hansson V Oestrogen receptors in the rat adrenal gland. J Steroid Biochem (1978) 9:957–62. 10.1016/0022-4731(78)90057-2 745396

[57] Calandra RS Purvis K Naess O Attramadal A Djoseland O Hansson V Androgen receptors in the rat adrenal gland. J Steroid Biochem (1978) 9:1009–15. 10.1016/0022-4731(78)90057-2 745391

[58] Calandra RS Luthy I Finocchiaro L Terrab RC Influence of sex and gonadectomy on sex steroid receptors in rat adrenal gland. J Steroid Biochem (1980) 13:1331–5. 10.1016/0022-4731(80)90094-1 6969821

[59] Bentvelsen FM McPhaul MJ Wilson CM Wilson JD George FW. Regulation of immunoreactive androgen receptor in the adrenal gland of the adult rat. Endocrinology (1996) 137:2659–63. 10.1210/en.137.7.2659 8770883

[60] van Lier E Carriquiry M Meikle A Sex steroid modulation of cortisol secretion in sheep. Animal (2014) 8:960–7. 10.1017/S1751731114000780 24703387

[61] Schutze S Wiegmann K Machleidt T Kronke M. TNF-induced activation of NF-kappa B. Immunobiology (1995) 193:193–203. 10.1016/S0171-2985(11)80543-7 8530143

[62] Aggarwal BB. Signalling pathways of the TNF superfamily: a double-edged sword. Nat Rev Immunol (2003) 3:745–56. 10.1038/nri1184 12949498

[63] Wajant H Pfizenmaier K Scheurich P. Tumor necrosis factor signaling. Cell Death Differ (2003) 10:45–65. 10.1038/sj.cdd.4401189 12655295

[64] Lesniewska B Miskowiak B Nowak M Malendowicz LK Sex differences in adrenocortical structure and function. XXVII. The effect of ether stress on ACTH and corticosterone in intact, gonadectomized, and testosterone- or estradiol-replaced rats. Res Exp Med (Berl) (1990) 190:95–103. 10.1007/PL00020011 2161554

[65] Provencher PH Tremblay Y Belanger A. Effects of C19 steroids on adrenal steroidogenic enzyme activities and their mRNA levels in guinea-pig fasciculata-glomerulosa cells in primary culture. J Endocrinol (1992) 132:269–76. 10.1677/joe.0.1320269 1311748

[66] Nowak KW Neri G Nussdorfer GG Malendowicz LK. Effects of sex hormones on the steroidogenic activity of dispersed adrenocortical cells of the rat adrenal cortex. Life Sci (1995) 57:833–7. 10.1016/0024-3205(95)02015-B 7630311

[67] Rossi R Zatelli MC Valentini A Cavazzini P Fallo F del Senno L Evidence for androgen receptor gene expression and growth inhibitory effect of dihydrotestosterone on human adrenocortical cells. J Endocrinol (1998) 159:373–80. 10.1677/joe.0.1590373 9834454

[68] Yanes LL Romero DG. Dihydrotestosterone stimulates aldosterone secretion by H295R human adrenocortical cells. Mol Cell Endocrinol (2009) 303:50–6. 10.1016/j.mce.2008.12.020 19428991 PMC2681414

[69] Caroccia B Seccia TM Campos AG Gioco F Kuppusamy M Ceolotto G GPER-1 and estrogen receptor-beta ligands modulate aldosterone synthesis. Endocrinology (2014) 155:4296–304. 10.1210/en.2014-1416 25167221

[70] Yoshimura S Sakamoto S Kudo H Sassa S Kumai A Okamoto R. Sex-differences in adrenocortical responsiveness during development in rats. Steroids (2003) 68:439–45. 10.1016/S0039-128X(03)00045-X 12798494

