# Supplementary figures and images for: Transcriptome Profile of Rat Adrenal Evoked by Gonadectomy and Testosterone or Estradiol Replacement
Source: Front Endocrinol (Lausanne). 2017 Feb 15;8:26. doi: 10.3389/fendo.2017.00026 (PMC5309227; doi:10.3389/fendo.2017.00026)

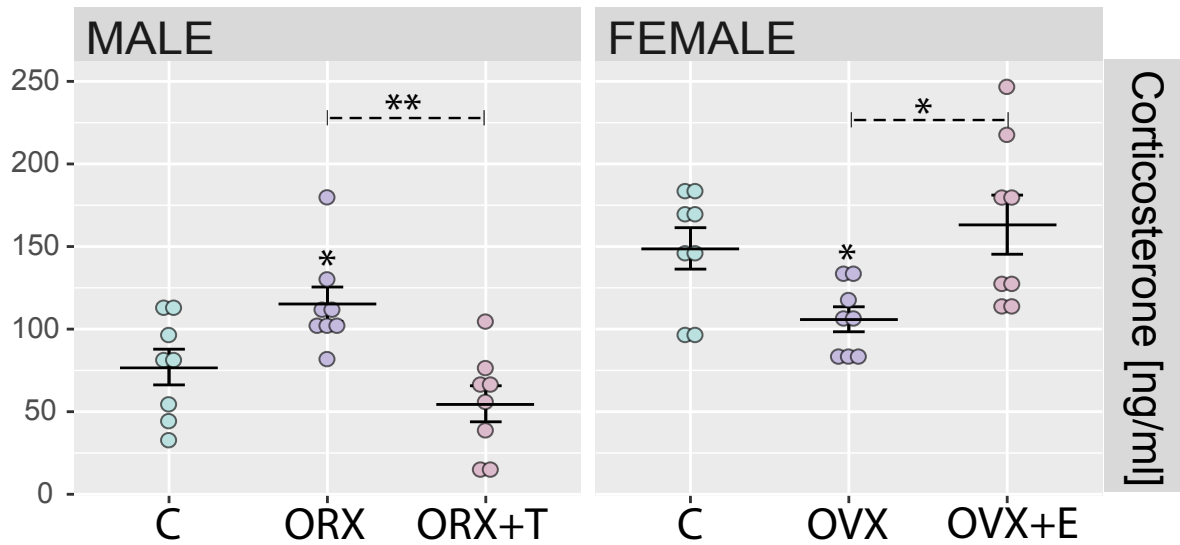

Supplement: Figure S1 — Serum corticosterone concentrations in adult male and female control, gonadectomized and gonadal hormone replaced rats. Bars represent mean ± SEM. Each circle represents an individual value. Statistically significant differences in relation to control or gonadectomized group: *p < 0.05; **p < 0.02; ***p < 0.01; ****p < 0.001. [file image_1.pdf]

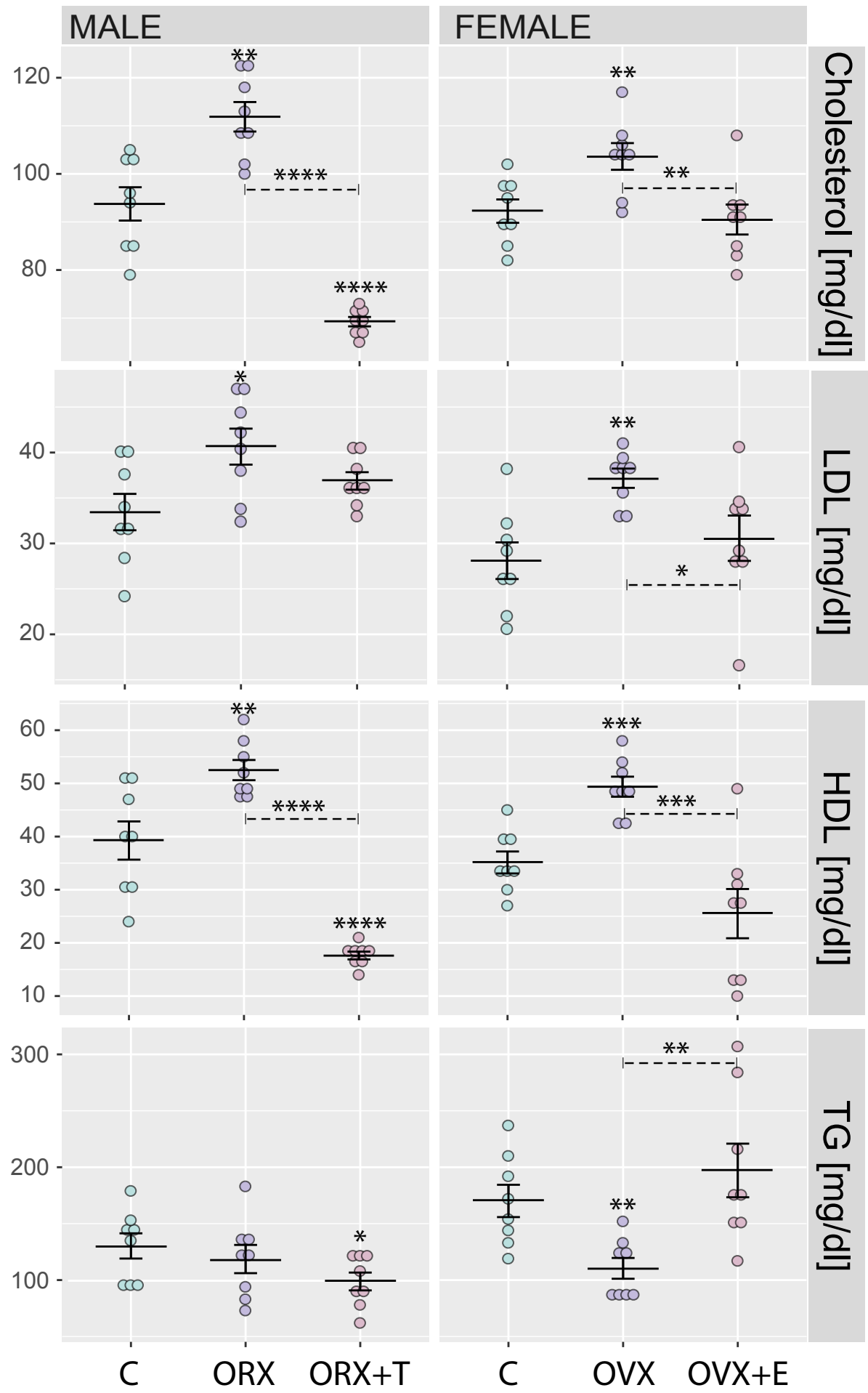

Supplement: Figure S2 — Serum total cholesterol, LDL, HDL, and triglyceride concentrations in adult male and female control, gonadectomized and gonadal hormone-replaced rats. Bars represent mean ± SEM. Each circle represents an individual value. Statistically significant differences in relation to control or gonadectomized group: *p < 0.05; **p < 0.02; ***p < 0.01; ****p < 0.001. [file image_2.pdf]
